# Instrumental Assessment and Pharmacological Treatment of Migraine-Related Vertigo in Pediatric Age

**DOI:** 10.3390/audiolres14010011

**Published:** 2024-01-29

**Authors:** Pasquale Viola, Alfonso Scarpa, Giuseppe Chiarella, Davide Pisani, Alessia Astorina, Filippo Ricciardiello, Pietro De Luca, Massimo Re, Federico Maria Gioacchini

**Affiliations:** 1 Unit of Audiology, Regional Centre of Cochlear Implants and ENT Diseases, Department of Experimental and Clinical Medicine, Magna Graecia University, 88100 Catanzaro, Italy; pasqualeviola@unicz.it (P.V.); davidepisani@unicz.it (D.P.); a.astorina@studenti.unina.it (A.A.); 2 Department of Medicine and Surgery, University of Salerno, 84084 Salerno, Italy; ascarpa@unisa.it; 3 Ear, Nose, and Throat Unit, AORN Cardarelli, 80131 Napoli, Italy; filippo.ricciardiello@aocardarelli.it; 4 Otolaryngology Department, San Giovanni-Addolorata Hospital, Via dell’Amba Aradam, 8, 00184 Rome, Italy; pietro.deluca.fw@fbf-isola.it; 5 Ear, Nose, and Throat Unit, Department of Clinical and Molecular Sciences, Polytechnic University of Marche, Via Conca 71, 60020 Ancona, Italy; m.re@univpm.it (M.R.); federicomaria.gioacchini@ospedaliriuniti.marche.it (F.M.G.)

**Keywords:** children, vertigo, dizziness, migraine, benign paroxysmal vertigo of childhood

## Abstract

Background: The most frequent form of vertigo in pediatric age is represented by vertigo linked to migraine, with a prevalence of 32.7%. This group of pathologies has received a redefinition of the diagnostic criteria to adapt them to the pediatric age with a new classification of the clinical pictures. We have several kinds of problems with these conditions that often have a significant impact on patients’ and parents’ quality of life: the diagnostic approach involves different tools for the different age groups contained in the pediatric range; the treatment of this type of vertigo is not consolidated due to the limited availability of trials carried out on pediatric patients. Focusing on this topic, the aim of this review was to provide an update on the more recent clinical advances in the diagnosis and treatment of Vestibular Migraine (VM) in children. Methods: We searched the PubMed, Embase, and Cochrane library databases for articles published in English from January 2015 to April 2023. The secondary search included articles from reference lists, identified by the primary search. Records were first screened by title/abstract, and then full-text articles were retrieved for eligibility evaluation. The searches combined a range of key terms (“Pediatric” AND “Childhood” AND “dizziness” OR “vertigo” AND “vestibular”). Results: Migraine-related vertigo, in its most recent definitions and classifications, is the most frequent group of balance pathologies in pediatric age. The results from the various experiences present in the literature suggest a clinical approach to be integrated with the use of instrumental tests selected according to the age of the patient and the reliability of the results. Conclusion: Knowing the timeline of the applicability of vestibular tests and the information that can be obtained from them is fundamental for diagnostic accuracy. Therapy is strongly conditioned by the limited availability of pediatric trials and by the wide range it includes, from very young children to adolescents.

## 1. Introduction

The prevalence of vestibular alterations in children is lower than in adults and ranges between 0.7% and 15% [1,2]. Vertigo in childhood is characterized by brief and vague symptoms. Furthermore, neural plasticity leads to better vertigo tolerability, with a self-limiting clinical history [3]. The differential diagnosis of childhood vestibular impairment also differs from adults because many pathological entities appear only in the pediatric age. Overall, the etiology of these alterations may comprise various peripheral and central vestibular disorders [4]. There are many concerns about the accuracy of the diagnosis of vertigo in children, related to the brief duration of the symptoms, parents’ underestimation, and the difficulty in obtaining direct information from the patient, which makes many pediatricians quite unaware about the correct management [5]. So, an exhaustive clinical and instrumental work-up is always necessary to achieve the correct diagnostic framework [6].

Despite significant technological improvements in diagnostics, the final diagnosis is still primarily based on patient history and physical examination [4]. Younger children find it difficult to easily explain what they are experiencing. So, it is important to ask parents to carefully describe each episode of vertigo. Possible associated symptoms, including pallor, sweating, vomiting, and behavioral attributes such as screaming, lying face down in the cot, and showing reluctance to be moved, should also be asked about [7].

Children with vestibular alterations are examined at the same time by different types of specialists, including pediatricians, psychiatrists, neurologists, otolaryngologists, and ophthalmologists. This may cause a high number of prescriptions for useless and expensive testing, often without obtaining pathognomonic information [3]. However, deciding which test to perform and the need for age-appropriate adaptations are critical due to the relatively short attention span of children. When vestibular testing is needed in a child, it is important to mention that not all tests are essential or appropriate for all children, depending on their age [8]. Furthermore, it is very important to test vestibular function in children with hearing loss, especially if they will have to undergo cochlear implantation (CI). In fact, this procedure is at risk for otolith damage because of the saccule’s proximity to the insertion pathway of the implant’s electrode array. It is estimated that between 40 and 80% of children have absent cervical vestibular evoked myogenic potential (cVEMP) responses, following CI [9,10]. More recently, a middle- to long-term evaluation of the vestibular function in CI patients, implanted in childhood, pointed out that 85% of patients were asymptomatic, with a mean time of >10 years after surgery. Vestibular impairment and symptoms seemed to be mainly due to the underlying inner ear’s disease rather than surgery [11].

In particular, the most frequent clinical picture is represented by vertigo linked to migraine. In a recent review, Vestibular Migraine (VM) and other migraine variants sum up to 32.7% [2]. This group of vertigo linked to migraine has received a redefinition of the diagnostic criteria to adapt them to the pediatric age, with a new classification of the clinical pictures. Focusing on this topic, the aim of this review was to provide an update on the more recent clinical advances in the diagnosis and treatment of Vestibular Migraine (VM) in children.

## 2. Materials and Methods

Two researchers (P.V. and F.M.G.) independently conducted literature research on PubMed, Embase, and Cochrane library databases for articles published from January 2015 until April 2023. The secondary search included articles from reference lists, identified by the primary search. Records were first screened by title/abstract, and then full-text articles were retrieved for eligibility evaluation. The searches combined a range of key terms (“Pediatric” AND “Childhood” AND “dizziness” OR “vertigo” AND “vestibular”) as well as their possible combinations. Only articles in the English language were considered. Any type of study (CRTs, meta-analyses, and systematic reviews) was included if it discussed vestibular testing and pharmacological treatment in pediatric age. All review articles were also read in full. The related citation tool in PubMed was used to search for further potential articles. References in all selected articles were also examined to identify additional relevant publications and ensure all applicable literature was included. Articles in French, Spanish, Chinese, or other languages were excluded. Case reports, short communications, letters, opinions, and editorials were excluded.

## 3. Results

**Figure 1:**
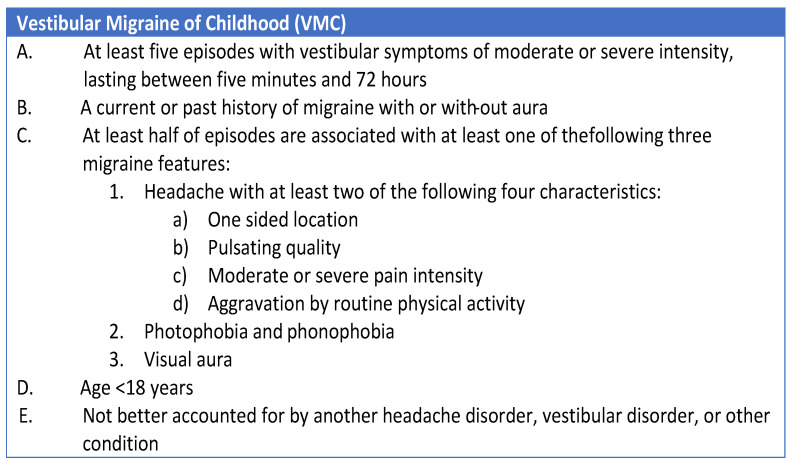
Diagnostic criteria for Vestibular Migraine in childhood [12].

**Figure 2:**
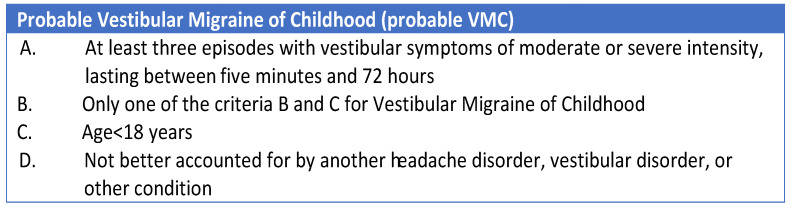
Diagnostic criteria for Probable Vestibular Migraine in childhood [12].

**Figure 3:**
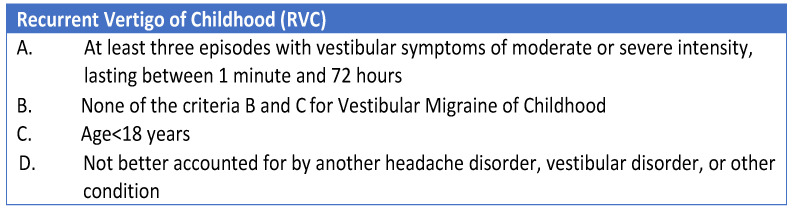
Diagnostic criteria for Recurrent Vertigo in childhood [12].

The most significant innovation on this topic was introduced in 2021 by van de Berg et al. [12], who proposed a new series of diagnostic criteria for “Vestibular migraine” and “Benign Paroxysmal Vertigo of Childhood”. The authors published a consensus document aimed at classifying “Vestibular Migraine of Childhood” (VMC) and “Probable Vestibular Migraine of Childhood” (PVMC), as well as introducing a new term and classification of recurrent vertigo in children, named “Recurrent Vertigo of Childhood” (RVC), which replaces the term “Benign Paroxysmal Vertigo of Childhood” (Figure 1, Figure 2 and Figure 3).

About this topic, most of the available literature and also our previous work from 2014 refer to the previous nosological classification. In the discussion, we will comment on the changes generated by this new consensus [13]. Certainly, the approach to these clinical pictures is made clearer. The first interesting data deriving from this new classification concerns RVC. Dunker et al., in their recent study, tried to better characterize clinical and instrument-based findings in patients with RVC and to evaluate the course of this disorder. Overall, 42 children (24 male and 18 female) with RVC were included. Attack duration ranged between 1 min and 4 h. The most common accompanying symptoms included nausea, vomiting, expressions of fear, and falls. Interestingly, female patients showed a higher age at symptom onset, attack frequency, and duration [14].

### 3.1. Recent Attempts to Perform an Etiological Classification

There is still a lack of a definitive classification in consideration of the etiology and incidence of childhood vestibular symptoms. Since our attempt to improve this aspect in 2014, only a few authors have achieved a valid upgrade to our work. Following this, we mention the most representative studies that can be found in the recent literature. Davitt et al. published a systematic review in 2017 including a total of 2726 children aged 2 months to 19 years. The main diagnosis associated with childhood vertigo was VM (23.8%; credible interval, 22.3–25.5%), while labyrinthitis/vestibular neuronitis were less frequent (8.47%; credible interval, 7.46–9.55%) [15]. In 2021, Wang et al. performed a retrospective cohort study analyzing a wide population of 1021 patients with vestibular alterations. Interestingly, among the total number of patients, 624 were female and 397 were male. The most common diagnosis resulted in VM (35.0%) [16]. In the same year, Fancello et al. reported that Vestibular Migraine (VM) and other migraine variants sum up to 32.7%, followed by audio-vestibular disorders (23.9%), psychogenic vertigo (11.3%), neurological diseases (10.4%), and post-traumatic vertigo (8.8%). Less frequent diagnoses (<5%) include motion sickness, cardiovascular diseases, and ophthalmic disorders [2]. In this regard, it is worth mentioning four episodic syndromes, including cyclic vomiting syndrome, abdominal migraine, benign paroxysmal vertigo, and benign paroxysmal torticollis, as well as other disorders such as infantile colic, alternating hemiplegia of childhood, acephalgic migraine, and acute confused migraine that may be entities on the migrainous spectrum [17]. Regarding possible differential diagnoses, it is worth mentioning episodic ataxia 2 in childhood, which manifests itself with episodes of ataxia (hours) with interictal nystagmus and, in some cases, with comorbid migraine [18]. In 2022, Urbančič et al. retrospectively evaluated the records of 257 children: 12.5% had peripheral vertigo, and 19% had central vertigo and dizziness. Acute vestibulopathy was diagnosed in 8.5% of children, and sudden sensorineural hearing loss and benign paroxysmal positional vertigo were diagnosed in 2% of children. Furthermore, 60% of children had peripheral vertigo, dizziness, and emesis, and 55.6% had nausea [19]. In 2023, Zhang et al. published an accurate systematic review that contributed to increasing knowledge about the main pathological entities that may cause vestibular symptoms in young subjects. Sinusitis-related diseases accounted for 10.7% of all vestibular symptoms in children, while VM was the most frequent etiological diagnosis associated with central vertigo (20.3%). In this paper, females appear more likely to experience psychogenic vertigo and VM. This phenomenon should be mainly explained by the influence of estrogen and the menstrual cycle in adolescent girls [20].

### 3.2. Vestibular Function Test in the Diagnosis of Children Vertigo

The diagnostic evaluation of pediatric vertigo includes a detailed medical history and a physical examination, as well as instrumental tests based on clinical indications appropriate for the age of the child [4]. It is important to understand which children should undergo diagnostic tests: children with a temporary hearing lesion or with various forms of vertigo, with coordination disorders or delayed development, and with learning difficulties. Instrumental vestibular test batteries may include the cervical and ocular Vestibular-Evoked Myogenic Potentials (cVEMPs, oVEMPs), the video Head-impulse Test (vHIT), and the bithermal Caloric Test (Fitzgerald–Hallpike test) (CT).

VEMPs evaluate utricular and saccular otolith functions. Early development of the vestibular colic reflex makes it possible to use cVEMPs testing in children younger than 12 months. The vHIT quantifying vestibulo-ocular reflex (VOR) function provides information on all semicircular canals (SC) and on each branch of the vestibular nerve at physiologically high frequencies (2–5 Hz). CT only assesses the function of the horizontal SC and superior branch of the vestibular nerve at ultralow frequencies (0.002 Hz) [21,22,23]. The vestibular system is both anatomically complete and functionally responsive at or before birth [24]; however, it develops until the end of puberty [25].

Vestibular tests should be chosen according to the child’s age (Table 1). Between 0 and 2 years, cVEMPs and vHIT with a remote system seem to be the most appropriate tests; between 3 and 7 years, the most suitable tests are vHIT, cVEMPs, and oVEMPs; after the age of 8, vHIT, CT, cVEMPs, and oVEMPs are applicable [26,27].

Wang et al. determined that repeatable cVEMP responses could be elicited in term (72%) and preterm (26%) infants within 5 days from birth if they reached a weight of 2.82 kg and 2.26 kg, respectively [28]. Regarding preterm infants, related to vestibular system maturation, cVEMPs may show prolonged and/or absent results [29,30]. The main cause of prolonged and/or absent cVEMPs is the myelination process, reflecting incomplete maturity of the sacculo-collic reflex [31,32]. Zhang et al. investigated the possible role played by CT and vHIT in VMC, PVMC, and RVC. The authors found dissociate results of CT and vHIT in 81 patients aged 5–17 years, with VMC (25.80%), PVMC (13.58%), and RVC (50.62%). CT and vHIT might occasionally produce conflicting results, particularly in peripheral vestibular disorders, with normal results only in one of the two tests. The abnormal CT rates in VMC, PVMC, and RVC patients were 24.14%, 36.36%, and 17.07%, respectively (*p* > 0.05). None showed abnormal vHIT results. The abnormal CT rates were significantly higher than those of the abnormal vHIT rates (*p* < 0.05). Based on their results, the authors concluded that neither CT nor vHIT are specific enough to clarify the diagnosis in these three conditions. CT appears to be more sensitive for diagnosing vestibular disease [33]. These tests evaluate SC at different frequencies, and, consequently, CT and vHIT associations allow for the global evaluation of the vestibular system [34,35,36]. Langhagen et al. found abnormal CT findings in 21.0 of RVC and 25.0% of VMC patients [37]. Marcelli et al. studied 22 children suffering from Vestibular Migraine (group A) and 18 children suffering from migraine without vestibular symptoms, aged between 8 and 13 years (group B). The authors found brainstem abnormalities in children with migraine as direct involvement of the peripheral or central vestibular pathways, or both. Some children with migraine without vestibular symptoms have also reported abnormalities on vestibular tests. This may demonstrate subclinical involvement of the vestibular pathways without clinical presentation [38]. An interesting aspect from the point of view of instrumental diagnosis concerns the value of the vHIT. Chen et al. compared the results of using this test in the diagnosis and evaluation of treatment results in a group of 36 patients with RVC versus controls. The comparison showed no statistically significant differences in vHIT alterations between the two groups. Demonstrating the variety of vestibular alterations in these patients, abnormalities were detected in 25.0% (reduced gains, response asymmetry, and isolated evident saccades). Only in the horizontal channels was a significant statistical result highlighted between these two groups. The lack of significance in the other cases may depend on the greater high-frequency participation in the vestibule in the daily activities of young patients, for which the high-frequency vestibular function being particularly prone to compensation would explain the reduced rate of anomalous vHIT results [39]. When using this method in the pediatric population, practicality of use is important. For this purpose, the use of a remote camera such as the Ulmer vHIT Synapsis^®^ device (Marseille, France) could be very useful. In this vHIT system, the camera that detects eye movements is positioned 1 m from the patients. Younger children can easily be studied while sitting on their parents’ laps. During head movements, the visual target can be represented by an illuminated toy or a cartoon presented by a monitor or a smartphone, or even by a ball [40]. This remote setup allows the vHIT to be used from 3 months of age, while the other systems can be used from 3 years of age [41]. It is important to mention that the gain values are lowest under 3 years of age, after which there is a rapid increase up to the age of 6 years, with a subsequent slower increase in vHIT gain up to 16 years [41,42]. It is worth mentioning that in some studies, the gain in vHIT was found to be stable from 4 to 18 years [43]. Similarly, in other experiences between older children and young adults, no significant differences in VOR gain values are reported [44,45]. The vHIT compared to CT does not induce vertigo, and vision is not occluded; for this reason, it is therefore more acceptable to children, and communication is smoother, especially in children with hearing loss. It is performed in about 15 min, regardless of the middle ear pathology. Possible vHIT disadvantages include inability to follow directions correctly, frequent blinking, wandering gaze, reduced alertness, and fear of receiving head impulses [44,46]. CT is performed with the child supine and head raised about 30 degrees and lasts about 20–25 min. Irrigations of cold (30 °C) and warm (44 °C) water or air are performed in each external ear canal, usually for 30 s for water and 60 s for air. Electrodes or infrared goggles are used to record eye movements during irrigation and for 60 s after irrigation. CT has been reported in infants as young as 2 months of age; even in newborn babies, it can be performed, but it is very stressful for babies and examiners. However, it is considered reliable from after 6–12 months of age, related to the child’s weight, and in clinical practice, it is used in children aged at least 6 years. CT can be frightening for children due to blocked vision; it also causes dizziness, hearing is temporarily impaired, and children are required to remain still for several minutes during and after the test. Another limitation is that CT cannot be used in the presence of middle ear pathology [47,48].

In the clinical-anamnestic evaluation, any alarm signals that point towards central causes must be identified, and, therefore, neuroimaging must be carried out to identify or exclude more serious causes. An MRI brain study is the most effective choice [49]. Furthermore, it could be of great help to educate parents to record acute events with cell phones or with devices that may record nystagmus with fixation block, especially when the attacks are brief.

### 3.3. Better Options in the Pharmacological Treatment for Specific Pediatric Vertigo

Few studies have been published concerning the efficacy of antimigraine drugs on the vestibular symptoms of VMC. There is currently no evidence-based approach to VMC treatment; most indications are extrapolated from adult migraine studies, and even in adults, open randomized controlled trials are scarce. Often, most experiences are related to headaches and not always to dizziness. Vestibular suppressants and triptans are used during the acute phase in children and adolescents, although their effectiveness is yet to be verified [50,51]. VMC and RVC prophylaxis is indicated when the attacks are more than three per month or if the symptoms are severe using the same migraine drugs [52,53]. Drugs that are used for prophylactic purposes are cyproheptadine (2–4 mg/day), flunarizine (5–10 mg/day), propranolol (40–80 mg/day), and topiramate (1–2 mg/kg/day) [54]. They are naturally chosen in relation to individual parameters such as age, body mass index, any comorbidities, possible side effects, and compliance of the patient and caregivers. The result of a retrospective analysis on 28 patients (9–18 years) is interesting and documented how pharmacological prophylaxis in children and adolescents can intervene on the frequency and severity of positive episodes of VM. Among the drugs used and studied, tricyclic antidepressants and cyproheptadine showed the best results, with improvements of over 85%; topiramate and triptans achieved improvements of 80%; and the use of gabapentin was much less satisfactory. For the treatment of the acute phase, vestibular suppressant drugs (i.e., meclizine, prochlorperazine, promethazine, ondansetron, and scopolamine) have been used with unsatisfactory results [51]. With reference to tricyclics, it is important to underline here the lesser manageability of these drugs in the pediatric population due to the possible side effects, which include blurred vision, palpitations, drowsiness, and arrhythmia [55]. In a comparative study, flunarizine and topiramate were reported to have comparable efficacy [56]. In reality, the experience of Tekim and Edem reports that treatment with topiramate achieved a greater frequency of attacks compared to flunarizine [54]. Currently, pharmacological treatment should not be considered the first therapeutic choice for pediatric patients. Treatment includes riboflavin, a healthy lifestyle with a healthy diet, adequate sleep, and limited use of technology [53]. Vestibular rehabilitation is useful in VM in the same way as cognitive behavioral therapy and biofeedback, useful as first-line therapy and also to prevent migraine [57,58,59]. BPV is a self-limiting disease; management, therefore, relies on the reassurance of children and their parents. In general, vertigo in children is well controlled, the autonomic component is minor, and affected children tend to return to normal daily activities more quickly than adults [60].

## 4. Discussion

During the publication of our previous systematic review in 2014 [13], we found some difficulties in describing the clinical differences intercurrent between “Vestibular Migraine” and “Benign Paroxysmal Vertigo of Childhood”. In our opinion, this new classification represents valid support and should be widely adopted in clinical practice because it may help to categorize three distinct pathological entities based on well-defined clinical criteria. It is also probable that, with the systematic application of these new diagnostic classifications, some interesting data will be obtained about these three specific conditions.

Having therefore established that, from an epidemiological point of view, VM-related vertigo is the most frequent form in the pediatric age, it becomes particularly interesting to understand how it is distributed in the various ages of the broad pediatric range. Fancello et al. [2] analyzed the frequency of different diagnoses in relation to three age groups: for the migraine group, preschool (0–5 years old) was 60.2%, elementary school (6–11 years old) was 54.4% and adolescents (12–18 years old) were 44.9%. This brings us directly to the need to know which diagnostic tools can be applied to these very different age groups. For this purpose, we have reported what the literature suggests on which tests are applicable and what information they can give us at different ages in the pediatric range. The specific consideration of the vertigo related to migraine is, however, that the diagnosis is primarily linked to the anamnestic and clinical data and only partly to the instrumental results. Finally, treatment represents a complex topic for all pediatric vertigo. The causes are to be identified in some well-known aspects of this period of life, which include the easy misrecognition of vestibular diseases, the limits of the anamnesis due to poor collaboration, the different prevalence of different diseases precisely in relation to age, and the risk of being faced with multiple diagnoses and overlaps. This makes dynamic management of these patients necessary and, especially for those with VMC, requires that treatment become a shared decision-making process. The approach could in fact be non-pharmacological, with the elimination of triggering factors, an adequate diet, good hydration, the introduction of adequate counseling, and psychological management. Good results can derive from physical activity and, if necessary, from vestibular rehabilitation. If the frequency or severity of the attacks makes it necessary, treatment can become pharmacological, and, in this regard, we have presented data that, however, is limited in childhood, with an effect mainly on headaches and not always on vertigo. Also, in this case, it is worth mentioning that many of the drugs usually used could be off-label due to indication or age.

## 5. Conclusions

Migraine-related vertigo, in its most recent definitions and classifications, is the most frequent group of balance pathologies in pediatric age. The results from the various experiences present in the literature suggest a clinical approach to be integrated with the use of instrumental tests selected according to the age of the patient and the reliability of the results. Knowing the timeline of the applicability of vestibular tests and the information that can be obtained from them is fundamental for diagnostic accuracy. Likewise, therapy is strongly conditioned by the limited availability of pediatric trials and by the wide range it includes, from very young children to adolescents.

## Figures and Tables

**Table 1:** Functional vestibular tests recommended in relation to the patient’s age.

Age	cVEMP	oVEMP	Caloric	vHIT
0–2	X			X
3–7	X	X		X
>8	X	X	X	X

## Data Availability

Not applicable.
